# Interaction of the O-Benzoyl-**β**-aminopropioamidoximes with Lawesson's Reagent and Spectral Characterization of the Products

**DOI:** 10.5402/2012/945893

**Published:** 2012-03-22

**Authors:** Lyudmila Kayukova, Kaldubai Praliyev, Ulan Kemelbekov, Asel Abdildanova, Vanda Gutyar

**Affiliations:** ^1^Laboratory of Synthetic and Natural Drug Chemistry, JSC “A. B. Bekturov Institute of Chemical Sciences”, 106 Shokan Ualickhanov Street, Almaty 050010, Kazakhstan; ^2^Laboratory of Physical Methods, Sh. Ualikhanov Kokshetau State University, 76 Abay Street, Kokshetau 020000, Kazakhstan

## Abstract

Interaction of O-benzoyl-*β*-aminopropioamidoximes [*β*-amino group: pyperidin-1-yl; morpholin-1-yl; thiomorpholin-1-yl; 4-phenylpiperazin-1-yl; benzimidazol-1-yl] with Lawesson's reagent was done in tetrahydrofuran at heating to 70°C during 10 h. New O-thiobenzoyl-*β*-aminopropioamidoximes were obtained with the outputs 57–96%; they were characterized with the help of physicochemical, IR, and NMR spectra.

## 1. Introduction

Representatives of thion-containing compounds, in particular, ethionamide and prothionamide are used in medicine as anti-TB drugs [1]. A high anti-HIV activity of thion derivative of dibenzoyl substituted piperazine was revealed; thion derivative gives 100% inhibition of the HIV virus in comparison with 11% inhibition of the original carbonyl compounds [2]. In our group samples with high antitubercular activity were revealed within the row of *β*-aminopropioamides [3–5]. For us it was interesting to investigate the effect of replacing the oxygen atom in carbonyl group on a sulfur atom on the biological properties of studied compounds, primarily on the antitubercular properties.

There are several methods for conversion of carbonyl compounds to thion contained; the main one is the interaction of carbonyl compounds with phosphorus pentasulfide P_4_S_10_ (reagent Berzelius) [6], as well as Lawesson's reagent [7]. Reaction with phosphorus pentasulfide requires high temperatures and large excess of this substance. Lawesson's reagent [2,4-bis (4-methoxyphenyl)-1,3-dithia-2,4-diphosphethane-2,4-disulfide] is a convenient thionation reagent for ketones, ethers, and amides which allows to conduct synthesis of corresponding thion analogs with good outputs. The reaction can be carried out in solvents (THF, benzene, toluene, dioxane, pyridine) at heating [7–12] or at exposition to microwave radiation on a neutral Al_2_O_3_ support at solvent free conditions [12–14]. The ratio of substrate—Lawesson's reagent can be varied from 1 : 0.5 up to 1 : 1. Herein we would like to report an efficient way of thion derivatives synthesis from O-benzoyl-*β*-aminopropioamidoximes in good yields.

## 2. Results and Discussion

**Scheme 1:**
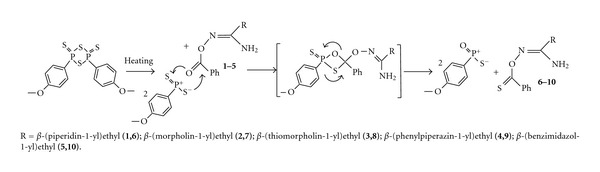


**Scheme 2:**
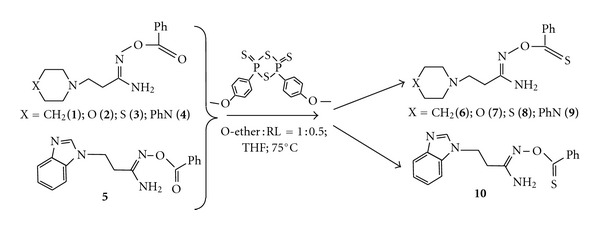


In this work the synthesis of several O-thiobenzoyl-*β*-aminopropioamidoximes [*β*-amino groups are piperidin-1-yl (**6**), morpholin-1-yl (**7**), thiomorpholin-1-yl (**8**), 4-phenylpiperazin-1-yl (**9**), benzimidazol-1-yl (**10**)] was performed at the interaction of O-benzoyl-*β*-aminopropioamidoximes with Lawesson's reagent in tetrahydrofuran with heating at 70°C during 10 h; the ratio of reactants was as 1 : 0.5. When heated, the disclosure of a central four-membered diphosphethane cycle of Lawesson's reagent occurs with the formation of two particles of thiophosphene ylide interacting with **1**–**5** as shown in Schemes 1 and 2.

**Scheme 3:**
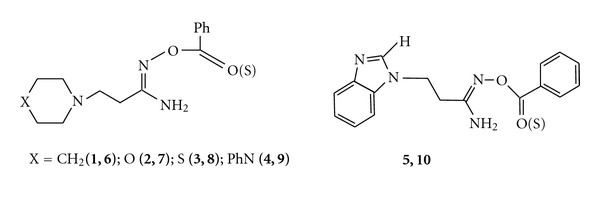


The reaction products were characterized by physicochemical data, IR, and NMR spectra (Tables 1–3 and Scheme 3).

In Table 2 the infrared spectra of O-ethers-*β*-aminopropioamidoximes (**1–5**) are given for comparison with the products spectra (**6**–**10**).

IR spectra of O-thiobenzoyl-*β*-aminopropioamidoximes (**6**–**10**) show disappearing of the bands of stretching vibrations of carbonyl groups *ν*_C=O_ of parent compounds (**1–5**) in the region 1717–1719 cm^−1^, when the bands of stretching vibrations of thion bonds are at *ν*_C=S_ 1290–1316 cm^−1^. Group of bands of stretching vibrations of N–H bonds (*ν*_N-H_) of the initial compounds (**1–5**) consisting of three bands above 3196 cm^−1^ became one broadband at *ν*_N-H_ 3300–3399 cm^−1^ in the spectra of the products (**6–10**). In addition the IR spectra of compounds (**6**–**10**) have a number of characteristic bands of stretching and bending vibrations: 1643–1688 cm^−1^ (*ν*_C=N_); 1568–1642 cm^−1^ (*δ*_H-N_ and *ν*_C=C_), 1249–1269 cm^−1^ (*ν*_C-O_); 1092–1133 cm^−1^ (*ν*_C-N_), 1025–1100 cm^−1^ (*ν*_N-O_) (Table 2).

Table 3 and Scheme 3 show the ^1^H NMR spectra of O-thiobenzoyl-*β*-aminopropioamidoximes (**6–10**); data of proton magnetic resonance for the initial O-ether-*β*-aminopropioamidoximes (**1**–**5**) are shown there for comparison. ^1^H NMR spectra signals of **6**–**10** indicate the preservation of the original structure of *β*-aminopropioamidoximes O-ethers. It should be pointed out that all signals of the thion derivatives **6**–**10** in different degrees are shifted to the lower fields in comparison with starting O-benzoyl-*β*-aminopropioamidoximes (**1–5**).

 Thus compounds **6**–**10 **in the resonance region of aromatic protons C_sp2_ H give signals at *δ* 6.80–9.25 ppm, whereas these signals of O-benzoyl groups in **1**–**5 **are at *δ* 6.77–8.62 ppm.

Proton signals of NH_2_ groups of **6**–**10** are situated in the area *δ* 6.87–6.97 ppm; analogous signals of **1**–**5 **in the region *δ* 6.54–6.87 ppm with the highest shift to the low field Δ*δ* 0.43 ppm for a pair of compounds **3** and **8**. 

Protons of *α*-methylene group of compounds **6**–**10** have a resonance at *δ* 2.69–3.15 ppm and compounds **1**–**5 **in the area *δ* 2.26–2.74 ppm with a maximum shift to the low field Δ*δ* 0.80 ppm for a pair of compounds **4** and **9**. *β*-Methylene protons of **6**–**10** give signals at *δ* 3.05–4.85 ppm and of **1**–**5** in the region *δ* 2.54–4.63 ppm with a maximum shift to the low field Δ*δ* 1.27 ppm for a pair of compounds **4** and **9**. 

Protons of methylene groups of *β*-heterocycles: (CH_2_)_3_ (**6**), as well as the protons attached to the heteroatom in the 4-position of compounds (**7–9**), have a resonance at *δ* 1.42 and 2.46 ppm and *δ* 3.73–3.93 ppm, respectively, while the similar protons of the oxygen analogues **1 **and **2**–**4 **at *δ* 1.37 and 1.50 ppm and *δ* 2.69–3.56 ppm with a maximum shift to low field Δ*δ* 1.26 ppm for a pair of compounds **3 **and **8**. 

Signals of methylene groups of heterocycles connected to the nitrogen atom (N1) – N(CH_2_)_2_ have the largest shifts to the low field: region *δ* 3.51–3.80 ppm (**6–9**) compared with the region *δ* 2.37–2.66 ppm (**1–4**) with the maximum shift to low field Δ*δ* 1.36 ppm which is characteristic for the pair of compounds **1 **and **6**. 

**Scheme 4:**
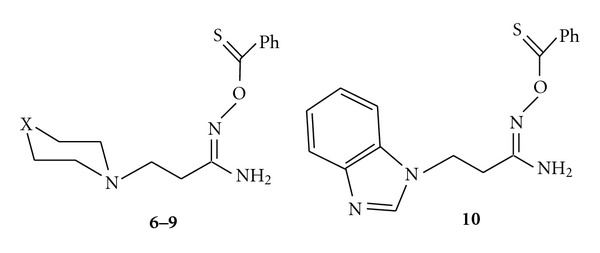


Obviously such descreening effect of thiocarbonyl sulfur atom in the last three groups of protons reflects the spatial structure of thion derivatives **6**–**10** where the sulfur atom may be closed to these descreened groups of protons in the syn-isomer with the s-trans conformation as shown in Scheme 4. 

A similar spatial structure with a syn-arrangement of N–O bond and the amino group NH_2_ about C=N bond with s-trans conformation of the substituents with respect to N–O linkage was found in the hydrochlorides of aroylation products of O-*β*-aminopropioamidoximes with the help of X-ray analysis [15]. 

## 3. Conclusion 

Thus, we have elaborated acceptable method of synthesis of O-thiobenzoyl-*β*-aminopropioamidoximes by using of interaction of O-benzoyl-*β*-aminopropioamidoximes with Lawesson's reagent at a ratio 1 : 0.5 at heating in THF at 70°C for 10 h. NMR spectral characteristics of new O-thiobenzoyl-*β*-aminopropioamidoximes confirm the structure established on the basis of elemental analysis and infrared spectra and provide proof of the spatial arrangement of functional groups in the s-trans-syn-configuration. 

## 4. Experimental Section 

IR spectra were recorded on a device NICOLET 5700 FT-IR in the range 400–3600 cm^−1^ in KBr pellets. ^1^H NMR spectra were taken on a JNN-ECA400 (400 MHz) spectrometer with HMDS as internal standard (*δ* 0.05 ppm); DMSO-d_6_ was used as a solvent. Monitoring of the reaction progress was carried out by thin layer chromatography (TLC) on Sorbfil plates (EtOH–benzene, 3 : 1, as the eluent). Solvents used for synthesis and recrystallization of the original *β*-aminopropionitriles, *β*-aminopropioamidoximes, O-aroyl- and O-thioaroyl-*β*-aminopropioamidoximes (EtOH, CHCl_3_, *i*-PrOH, THF), as well as solvents used for TLC (EtOH, benzene), were prepared according to standard procedures. 

Syntheses of starting *β*-aminopropionitriles, *β*-aminopropioamidoximes and O-benzoyl-*β*-aminopropioamidoximes (**1–5**) are given in [16–20].

***O-Thiobenzoyl-*β*-(piperidin-1-yl)propioamidoxime *(6, Table 1)**

Portionwise under cooling with ice water and stirring, Lawesson's reagent (1.38 g, 0.0035 mol) was added to the solution of O-benzoyl-*β*-(piperidin-yl)propioamidoxime (**1**) [16] (1.88 g, 0.007 mol) in 15 mL of dry THF. The reaction mixture was heated at 60–70°C through an hour of stirring at room temperature. After 1 h white precipitate of thiobenzoylation product (**6**) started to precipitate; it was heated for 9 h with TLC monitoring every hour and then product (**6**) was filtered off and dried. Recrystallization of the precipitate from CHCl_3_ gave 1.17 g (57%) of O-thiobenzoyl-*β*-(piperidin-1-yl)propioamidoxime (**6**) with *R*_f_ 0.82 and mp 170°C.

***O-Thiobenzoyl-*β*-(morpholin-1-yl)propioamidoxime *(7, Table 1)**

Portionwise under cooling with ice water and stirring, Lawesson's reagent (0.65 g, 0.0016 mol) was added to the solution of O-benzoyl-*β*-(morpholin-1-yl)propioamidoxime (**2**) [17] (0.89 g, 0.0032 mol) in 15 mL of dry THF. After 1 h of stirring at room temperature the reaction mixture was heated for 10 h at 60–70°C with TLC monitoring every hour. Through 1 h of heating a white precipitate of product **7** began to form; then at the end of reaction it was filtered off and dried. Recrystallization from EtOH gave 0.59 g (63%) of O-thiobenzoyl-*β*-(morpholin-1-yl)propioamidoxime (**7**) with *R*_f_ 0.70 and mp 168°C.

***O-Thiobenzoyl-β* *-(thiomorfolin-1-yl)propioamidoxime *(8, Table 1)**

Under cooling with ice water and stirring, portions (5 times) of Lawesson's reagent (0.47 g, 0.00115 mol) were added to O-benzoyl-*β*-(thiomorpholin-1-yl)propioamidoxime (**3**) [18] (0.66 g, 0.0023 mol) in 15 mL of dry THF. Turbidity of reaction mixture was forming after stirring at room temperature for about 1 h; then the reaction mixture was heated at 70°C during 10 hours with TLC monitoring. After 2 h of heating a white precipitate of **8** began to fall. At the end of the reaction **8** was filtered and recrystallized from EtOH. O-Thiobenzoyl-*β*-(thiomorpholin-1-yl)propioamidoxime (**8**) was gathered in amount 0.54 g, 75% with *R*_f_ 0.45; mp 217°C.

***O-Thiobenzoyl-*β*-(4-phenilpiperazin-1-yl)propioamidoxime *(9, Table 1)**

Under cooling with ice water, stirring portions of Lawesson's reagent (0.14 g, 0.00035 mol) were added to a solution of O-benzoyl-(*β*-4-phenilpiperazin-1-yl)propioamidoxime (**4**) [19] (0.2 g, 0.00056 mol) in 15 mL of dry THF. The reaction mixture was stirred at room temperature for an hour and heated at 70°C with TLC monitoring. Product **9** was isolated after 10 h of heating and recrystallization from CHCl_3_ (0.2 g, 96%; *R*_f_ 0,75; mp 130°C).

***O-Thiobenzoyl-(*β*-benzimidazol-1-yl)propioamidoxime* (10, Table 1)**

Portions of Lawesson's reagent (0.2 g, 0.005 mole) were added to a solution O-benzoyl-(*β*-benzimidazol-1-yl) propioamidoxime (**5**) [20] (0.3 g, 0.001 mol) in 15 mL of dry THF under cooling with ice water and stirring. The reaction mixture was heated at 70°C with TLC monitoring during 10 h; white precipitate of **10 **began to fall after 2 h of heating; it was isolated at the end of the reaction and recrystallized from CHCl_3_ (0.2 g, 62%; *R*_f_ 0,79; mp 126°C).

## Figures and Tables

**Table 1:** Physicochemical characteristics of O-thiobenzoyl-*β*-aminopropioamidoximes (**6–10**).

No.	*β*-Amino group	Output, %	*R*_f_	Mp/°C (solvent)	Found (%)			Molecular formula
					Calculated (%)			
					C	H	N	
**6**	Piperidin-1-yl	57	0.82	170 (CHCl_3_)	61.52	7.70	16.62	C_15_H_21_N_3_OS
					61.83	7.26	16.20	
**7**	Morpholin-1-yl	63	0.7	168 (EtOH)	57.59	6.60	14.03	C_14_H_19_N_3_O_2_S
					57.32	6.53	14.32	
**8**	Thiomorpholin-1-yl	75	0.45	217 (EtOH)	54.75	6.67	13.87	C_14_H_19_N_3_OS_2_
					54.34	6.19	13.58	
**9**	4-Phenylpiperazin-1-yl	96	0.75	130 (CHCl_3_)	65.65	6.23	15.61	C_20_H_24_N_4_OS
					65.20	6.60	15.20	
**10**	Benzimidazol-1-yl	62	0.79	126 (CHCl_3_)	62.75	4.67	17.70	C_17_H_16_N_4_OS
					62.94	4.97	17.27	

**Table 2:** IR-spectra of O-benzoyl- and O-thiobenzoyl-*β*-aminopropioamidoximes (**1–10**).

No.	*β*-Amino group	Stretching and bending bond vibrations, cm^−1^, KBr pellets							
		*ν*_ C=O_	*ν*_ C=N_	*δ*_ N-H_ and *ν*_C=C_	*ν*_ C=S_	*ν*_ C-O_	*ν*_ C-N_	*ν*_ N-O_	*ν*_ N(-H)2_
**1**	Piperidin-1-yl	1719	1637	1611	—	1277	1117	1070	3201; 3329; 3455
**2**	Morpholin-1-yl	1719	1637	1610; 1613	—	1280	1117	1070	3198; 3331; 3455
**3**	Thiomorpholin-1-yl	1717	1637	1613	—	1278	1104	1070	3196; 3311; 3457
**4**	4-Phenylpiperazin-1-yl	1718	1637	1601; 1618	—	1280	1100	1072	3200; 3324; 3454
**5**	Benzimidazol-1-yl	1717	1635	1612; 1618	—	1277	1069	954	3196; 3310; 3452
**6**	Piperidin-1-yl	—	1674	1568; 1600	1297	1268	1123	1072	3340
**7**	Morpholin-1-yl	—	1674	1600	1296	1269	1123	1072	3334
**8**	Thiomorpholin-1-yl	—	1675	1568; 1600	1296	1267	1123	1073	3340
**9**	4-Phenylpiperazin-1-yl	—	1688	1600; 1642	1290	1249	1133	1100	3300
**10**	Benzimidazol-1-yl	—	1643	1600	1316	1257	1092	1025	3399

**Table 3:** ^1^H NMR spectra of O-benzoyl- (**1–5**) and O-thiobenzoyl-*β*-aminopropioamidoximes (**6–10**).

		*δ*, ppm (*J*/Hz)					
No.	*β*-Amino group	N(CH_2_)_2 _(4H)	(CH_2_)_3_ (**1**, **6**); O(CH_2_)_2 _(**2**, **7**); S(CH_2_)_2 _(**3**, **8**); PhN(CH_2_)_2_ (**4**, **8**)	Csp^2^H	*α*-CH_2 _(2H) (t)	*β*-CH_2 _(2H) (t)	NH_2_ (2H) (s)
**1 **	Piperidin-1-yl	2.37 m	1.37 m and 1.50 m (6H)	7.40–8.10 m (5 H)	2.26 (7.0)	2.54 (7.0)	6.58
**2 **	Morpholin-1-yl	2.40 t (4.5)	3.56 t (4.5) (4H)	7.49–8.11 m (5 H)	2.29 (7.2)	2.58 (7.2)	6.58
**3 **	Thiomorpholin-1-yl	2.66 m	2.69 m (4H)	7.23–8.11 m (5 H)	2.27 (7.0)	2.70 (7.0)	6.54
**4 **	4-Phenylpiperazin-1-yl	2.60 t (5.0)	3.12 t (5.0) (4H)	6.77–8.13 m (10 H)	2.35 (6.5)	2.66 (6.5)	6.61
**5 **	Benzimidazol-1-yl	—	—	7.62–8.12 m (9H); 8.62 s [1H; C(2)sp^2^H]	2.74 (7.0)	4.63 (7.0)	6.82
**6**	Piperidin-1-yl	3.73 m	1.42 m and 2.46 m (6H)	7.66–9.18 m (5H)	2.70 (7.0)	3.73 (7.0)	6.87
**7**	Morpholin-1-yl	3.51 m	3.73 m (4H)	7.45–9.16 m (5H)	2.69 (7.0)	3.05 (7.0)	6.87
**8**	Thiomorpholin-1-yl	3.80 t (7.0)	3.95 t (7.0)	7.50–9.25 m (5H)	2.79 (7.0)	3.15 (7.0)	6.97
**9**	4-Phenylpiperazin-1-yl	3.69 t (7.0)	3.93 t (7.0)	6.80–8.02 m (12 H)*	3.15 (7.0)	3.93 (7.0)	*
**10**	Benzimidazol-1-yl	—	—	7.83–8.33 m (11H)*; 8.90 s [1H; C(2)sp^2^H]	2.93 (7.0)	4.85 (7.0)	*

* Signal of NH_2_-group protons is in the field of aromatic protons signal at *δ* 6.60–8.02 ppm (**9**) and at *δ* 7.83–8.33 ppm (**10**).
